# Identification of the Hub Gene LDB3 in Stanford Type A Aortic Dissection Based on Comprehensive Bioinformatics Analysis

**DOI:** 10.1111/jcmm.70471

**Published:** 2025-03-18

**Authors:** Xinyi Liu, Xing Liu, Bin Wan, Yipeng Ge, Haiou Hu, Hong Yu, Meng Zhao, Huadong Li, Junming Zhu

**Affiliations:** ^1^ Department of Cardiovascular Surgery, Beijing Aortic Disease Center Beijing Anzhen Hospital of Capital Medical University Beijing China; ^2^ Department of Cardiovascular Surgery Xinqiao Hospital of Army Medical University Chongqing China; ^3^ Max Planck Insititute for Human Cognitive and Brain Sciences Leipzig Germany; ^4^ Institute of Neuroscience and Medicine (INM‐7: Brain and Behavior) Research Centre Jülich Jülich Germany; ^5^ Department of Otorhinolaryngology, Union Hospital, Tongji Medical College Huazhong University of Science and Technology Wuhan China; ^6^ Department of Physiology and Pathophysiology, School of Basic Medical Sciences Fudan University Shanghai China; ^7^ Department of Cardiovascular Surgery Union Hospital, Tongji Medical College Huazhong University of Science and Technology Wuhan China

**Keywords:** bioinformatics, differentially expressed genes, LDB3, single‐cell sequencing, Stanford type a aortic dissection, weighted gene coexpression network analysis

## Abstract

Stanford type A aortic dissection (TAAD) is a life‐threatening disease. This study explored the role of LIM domain binding 3 (LDB3) in TAAD progression. Four datasets from the Gene Expression Omnibus were analyzed to identify TAAD‐related hub genes. LDB3 single nucleotide polymorphisms (SNPs) were assessed in the UK Biobank. Western blotting and immunofluorescence detected LDB3 expression in angiotensin II (Ang II) stimulated human aortic vascular smooth muscle cells (HA‐VSMC), human samples, and a murine model. Bioinformatics identified tissue inhibitor of metalloproteinase‐1 (TIMP1) and LDB3 as TAAD hub genes. TIMP1 was expressed in macrophages, mesenchymal cells, and smooth muscle cells, while LDB3 was mostly expressed in smooth muscle cells. Validation showed TIMP1 was upregulated and LDB3 downregulated in TAAD. Six LDB3 SNPs were associated with aortic aneurysm and dissection in the UK Biobank. In human and murine samples, LDB3 expression was reduced in diseased tissues and co‐localized with smooth muscle. Ang II‐stimulated HA‐VSMC exhibited LDB3 reduction and altered intercellular connections. The aforementioned findings suggest that the newly identified gene LDB3 is crucial in the progression of TAAD.

**Abbreviations**
AD: aortic dissection
Ang II: angiotensin II
AUC: area under the curve
DEG: differentially expressed gene
ECM: extracellular matrix
EVG: Verhoeff–Van Gieson
FC: fold change
GEO: Gene Expression Omnibus
GO: Gene Ontology
HA‐VSMC: human aortic vascular smooth muscle cell
HE: haematoxylin–eosin
KEGG: Kyoto Encyclopedia of Genes and Genomes
LDB3: LIM domain binding 3
MMP: matrix metalloproteinase
PCs: principal components
PI3K/Akt: phosphatidylinositol 3‐kinase/protein kinase B
PVDF: polyvinylidene fluoride
scRNA‐seq: single‐cell RNA‐sequencing
SDS‐PAGE: sodium dodecyl sulfate‐polyacrylamide gel electrophoresis
SNPs: single nucleotide polymorphism
TAAD: Stanford type A aortic dissection
TIMP1: tissue inhibitor of metalloproteinase‐1
TIMPs: inhibitors of metalloproteinases
VSMCs: vascular smooth muscle cells
WGCNA: weighted gene coexpression network analysis

## 1 Introduction

Aortic dissection (AD) is a life‐threatening emergency leading to significant mortality, especially in patients with Stanford type A aortic dissection (TAAD) [1]. The aetiology of TAAD includes the creation of a false lumen in the aorta wall as a result of aortic intima rupture, which may spread further proximally or distally as the heart contracts and even rupture the artery wall [2]. The most effective treatment for TAAD is immediate operation with hybrid surgery [3]. At present, the treatment of TAAD remains very challenging due to the high complexity of surgical repair of the aorta and the occurrence of severe postoperative complications [4]. The surgical mortality rate remains high, from 17% to 26%, even in many large cardiac centers [5]. There is also no effective pharmacological treatment available for TAAD. Therefore, exploring the molecular and cellular basis of this entity is critical for the development of more effective treatments.

The amount of data generated via single‐cell and genome sequencing is growing rapidly as a result of technological advancements, giving us more powerful tools to better understand the underlying molecular biological systems. At the same time, the summary analyses available in different databases are also particularly important, as they can provide directions for future basic research.

We downloaded four datasets from the Gene Expression Omnibus (GEO) database for this study, combined them with weighted gene co‐expression network analysis (WGCNA) to screen and identify differentially co‐expressed genes, and then performed single‐cell RNA sequencing (scRNA‐seq) to evaluate the expression of differentially co‐expressed genes in TAAD cell clusters. Finally, we were able to successfully validate the expression of associated genes in human samples and in murine models.

## 2 Materials and Methods

### 2.1 Data Collection

**FIGURE 1:**
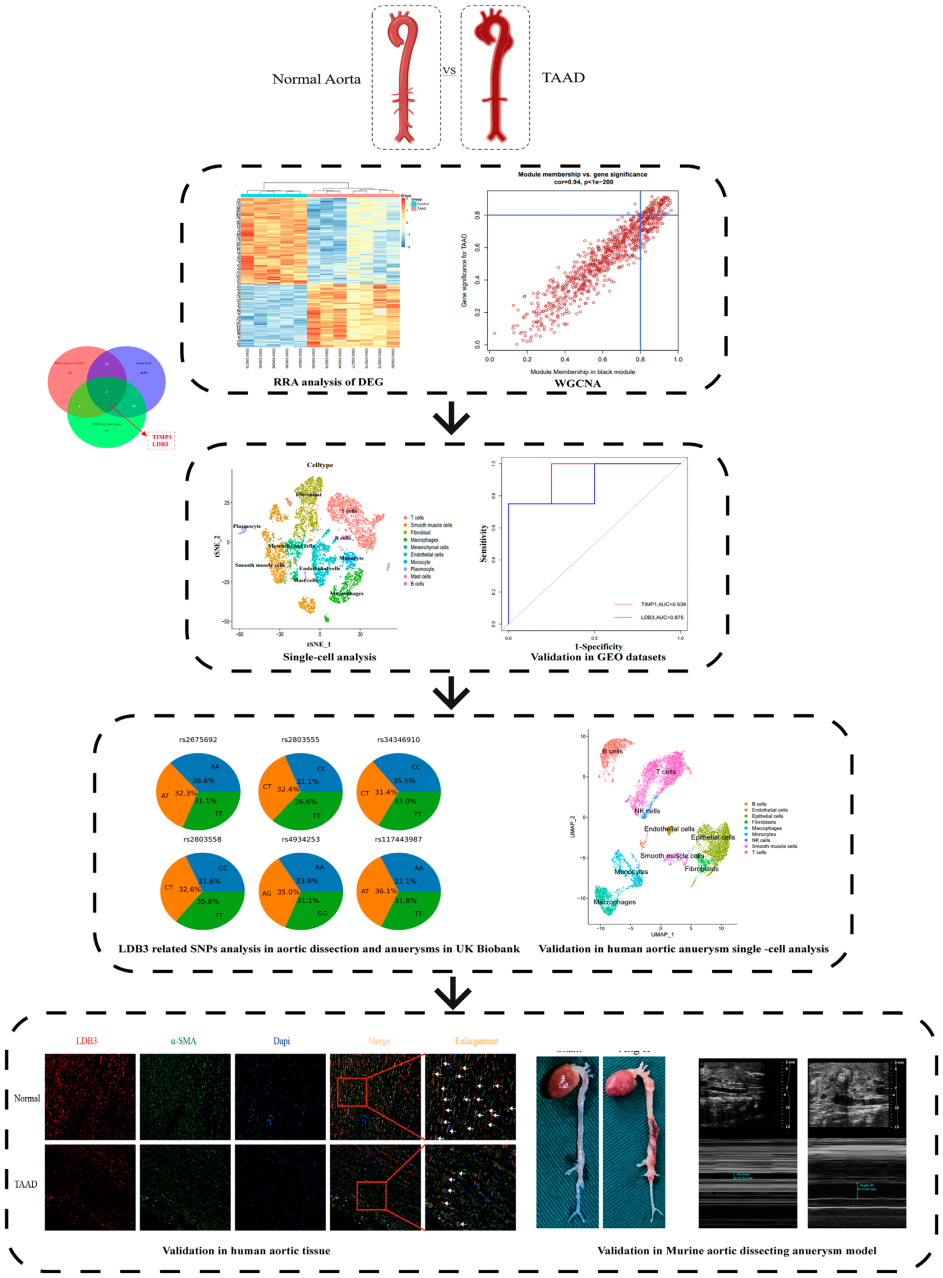
Flow chart of our study.

The study flow chart is shown in (Figure 1). Gene expression profiling data (GSE52093, GSE190635, GSE153434 and GSE213740) were downloaded from the GEO database (www.ncbi.nlm.nih.gov/geo/). GSE52093, GSE190635 and GSE153434 contain microarray data. GSE213740 contains scRNA‐seq data. GSE52093 is a dataset with seven disease samples and five control samples, and the data platform is GPL10558. The GSE190635 dataset, based on the GPL570 platform, includes four disease samples and four control samples. The GSE153434 dataset contains a total of 20 samples, 10 of which are control samples and 10 of which are TAAD samples, and the data platform is GPL20795 [6]. Six disease samples and three control samples are included in the scRNA‐seq dataset GSE213740, which is based on the GPL18573 platform [7]. The characteristics of the four datasets are shown in File S1. We downloaded information about 4249 genes related to TAAD from GeneCards (https://www.genecards.org/) (File S1).

### 2.2 Screening and Integration of DEGs

The affy package [8] in R was used to preprocess the original data for background correction and normalization. The R package limma [9] was used to screen the DEGs of datasets GSE52093 and GSE190635, with cutoffs set to *p* < 0.05 and |log fold change (FC)| > 0.5. The DEG volcano maps and heatmaps were drawn using the ggplot2 R package (https://ggplot2.tidyverse.org) and the pheatmap R package (https://CRAN.R‐project.org/package=pheatmap). The RRA R package (https://CRAN.R‐project.org/package=RobustRankAggreg) was used to integrate the DEGs of GSE52093 and GSE190635.

### 2.3 WGCNA in GSE153434

We performed WGCNA on the GSE153434 dataset with the WGCNA R package [10] using the ideal soft threshold of eight. The topological matrix was built using the soft threshold, and hierarchical clustering was conducted. Pearson correlation analysis was used to examine the associations between the modules and clinical characteristics, and the modules that were most positively associated with TAAD were discovered.

### 2.4 Single‐Cell Sequencing Data Preprocessing and Cell Type Annotation in GSE213740

The scRNA‐seq data from GSE213740 were preprocessed using the Seurat R package [11]. We identified cells expressing between 200 and 2500 genes and < 5% mitochondrial genes. The ‘FindVariableFeatures’ function in Seurat was used to determine the top 2000 hypervariable genes, and the ‘RunPCA’ function was used to determine 17 principal components (PCs). The ‘tSNE’ diagram was then used to depict the clustering of all cell clusters.

We identified DEGs in each cell cluster using the ‘FindMarkers’ function in Seurat. Then, according to the database ‘HumanPrimaryCellAtlasData’, the known role of the marker gene in the cell and the distance relationship between the cells in the ‘tSNE’ diagram were used to select the marker gene for the cell cluster. There were no appreciable discrepancies between the list of marker genes and those in the original research [7].

### 2.5 Pseudotime Trajectory Analysis

All objects annotated as smooth muscle cells were extracted and subjected to pseudotime analysis using the Monocle R package [12]. The ‘DDRTree’ function in the Monocle package was used to decrease the data dimensionality, and then the ‘reduceDimension’, ‘orderCells’ and ‘plot cell trajectory’ functions were used to determine the type of cell differentiation state and to show the cell differentiation trajectory.

### 2.6 Functional and Pathway Enrichment Analysis

Using the clusterProfiler R package [13], we performed Gene Ontology (GO) functional analysis and Kyoto Encyclopedia of Genes and Genomes (KEGG) pathway enrichment on the relevant DEGs, and a *p*‐value of 0.05 was established as statistically significant.

### 2.7 Variation of Single‐Nucleotide Polymorphisms (SNPs)

To establish the effects of genetic variation on TAAD, we analysed SNPs related to tissue inhibitor of metalloproteinase‐1 (TIMP1) and LIM domain binding 3 (LDB3) in the individuals who reported aortic aneurysm and dissection (data field: 131382) in the UK Biobank. First, we identified 19 SNPs of LDB3 on chromosome 10 that were previously reported to be associated with idiopathic dilated cardiomyopathy, including rs4256897, rs2675692, rs2803555, rs11812601, rs2803558, rs4468255, rs3740343, rs56165849, rs3740346, rs121908338, rs4934253, rs184737911, rs3802662, rs1986382, rs11202156, rs10887652, rs11202154, rs117443987 and rs34346901(doi: 10.1038/ejhg.2015.195). Both SNPs and questionnaire data were available for 487,186 individuals. Then, we performed univariate chi‐square tests to compare the rates of aortic aneurysm and dissection among the genotypes for each SNP. Eventually, forward stepwise logistic regression was conducted to associate multiple SNPs with aortic aneurysm and dissection. All the steps were performed in Python 3.8 with the Statsmodel package 0.13.2 and are available in our online IPython notebook.

### 2.8 Human Tissue

Ten aortic dissection tissues were obtained from patients diagnosed with Stanford TAAD who underwent open aortic dissection repair. Ten normal aortic tissues were obtained from patients undergoing heart transplantation. Twenty human samples were used for validation of target gene expression. The Union Hospital ethics committee approved this study. This study adheres to the World Medical Association Code of Ethics (Declaration of Helsinki), which was published in 1975. Detailed patient information is shown in (File S3).

### 2.9 Cell Culture and Treatment

During the progression of pathological processes in the aortic wall of patients with aortic dissection, the reduction of vascular smooth muscle cells (VSMCs) is a hallmark feature [14]. Angiotensin II (Ang II), a key effector of the renin‐angiotensin system, plays a critical role in the pathogenesis of aortic dissection [15]. To investigate whether changes in the expression of core genes are independent of the reduction in VSMC population and to further explore the detailed localisation of these crucial genes within VSMCs, we stimulated human aortic vascular smooth muscle cells (HA‐VSMCs) with Ang II. HA‐VSMC was purchased from the American Type Culture Collection (ATCC) and cultured with 20% fetal bovine serum (FBS) in smooth muscle cell medium (Sciencell, Catalogue #1101, USA). Ang II was purchased from Sigma (A9525; USA), and cells were incubated with 100 nM Ang II for 24 h before total protein was extracted and immunofluorescent staining.

### 2.10 Animal and Animal Models

All animal experiments were conducted in accordance with experimental protocols that were approved by the Animal Care and Use Committee at Tongji Medical College, Huazhong University of Science and Technology, China. The Apoe^−/−^ mice on a C57BL/6J background were obtained from The Jackson Laboratory (Stock No: 013225, USA) and housed in the Animal Center of Tongji Medical College. After anaesthesia with pentobarbital (50 mg/kg), mini‐pumps (Alzet osmotic pump model 1014D; USA) were sterilised and implanted subcutaneously with a delivery rate of 1000 ng/kg/min of Ang II (*n* = 10) (Sigma; A9525; USA) or saline (*n* = 5). After 4 weeks of implantation, aortic tissue was immediately obtained. Autopsies were performed on 2 mice (Ang II group) that died on day 10 and day 14 after implantation, and death was confirmed to result from thoracic aortic dissections. The remaining eight were obtained after 4 weeks’ treatment, and five of them found obvious arterial dissection or aneurysm.

### 2.11 Histology and Immunofluorescence Staining

The freshly collected human or murine aorta wall tissue was preserved with 4% paraformaldehyde. After being embedded in paraffin and sectioned (5 μm thick), the tissues were subjected to hematoxylin–eosin (HE), Verhoeff–Van Gieson (EVG) and Masson's trichrome staining and examined under a light microscope. For the EVG staining of mouse aortic tissues, we adopted the previous elastin degradation scoring criteria for quantitative analysis [16]. For immunofluorescence staining, in brief, prepared paraffin‐embedded sections were heated in a water bath, deparaffinized, subjected to antigen retrieval, cooled to room temperature, fixed, and blocked. Antibodies against α‐SMA (14395‐1‐AP; Proteintech, 1:100) and LDB3 (11004‐1‐AP; Proteintech, 1:100) were then used for staining. The specimens were observed under a confocal laser scanning microscope (Olympus, Tokyo, Japan).

### 2.12 Western Blotting

Protease inhibitors and phosphatase inhibitors were added to a 1:10 (w/v) ice‐cold solution that was used to homogenize the aortic samples. To obtain the supernatant, the lysate was centrifuged at 12,000 rpm (4°C) for 5 min. The BCA protein assay kit (P0010, Beyotime, China) was used to measure the protein quantities in the samples. The lysed samples were separated by using 10% sodium dodecyl sulfate‐polyacrylamide gel electrophoresis (SDS‐PAGE), and the separated samples were then transferred to polyvinylidene fluoride (PVDF) membranes. Anti‐LDB3 antibody (11004‐1‐AP; Proteintech, 1:1000) was incubated with the membranes at 4°C overnight after blocking with 5% skim milk for 2 h at room temperature. After three 5‐min rounds of washing, the PVDF membranes were incubated for 30 min at room temperature with HRP goat anti‐rabbit IgG (H + L) (AS014, ABclonal, 1:10,000). The PVDF membranes were next treated with ECL reagents (AS1059, ASPEN), rinsed four times in TBST for 5 min, and exposed to film. The protein bands were recorded using chemiluminescence detection devices (LiDE110, Canon, Japan). The grey intensity of the protein bands was analyzed by ImageJ software (NIH, Bethesda, MD).

## 3 Results

### 3.1 Screening and Integration of DEGs

A total of 2944 DEGs were screened from the GSE52093 dataset (File S4A,B), including 1512 upregulated genes and 1432 downregulated genes (File S5). The GSE190635 dataset contained 1163 DEGs (File S4C,D), including 358 upregulated genes and 805 downregulated genes (File S6). Using the RRA R package, the DEGs from the two datasets were integrated, and their sequences were retrieved. The code for RRA analysis is presented in (File S7). By using RRA analysis, we were able to generate 103 integrated DEGs, including 56 downregulated genes and 47 upregulated genes (File S8).

### 3.2 WGCNA and Identification of Hub Genes

**FIGURE 2:**
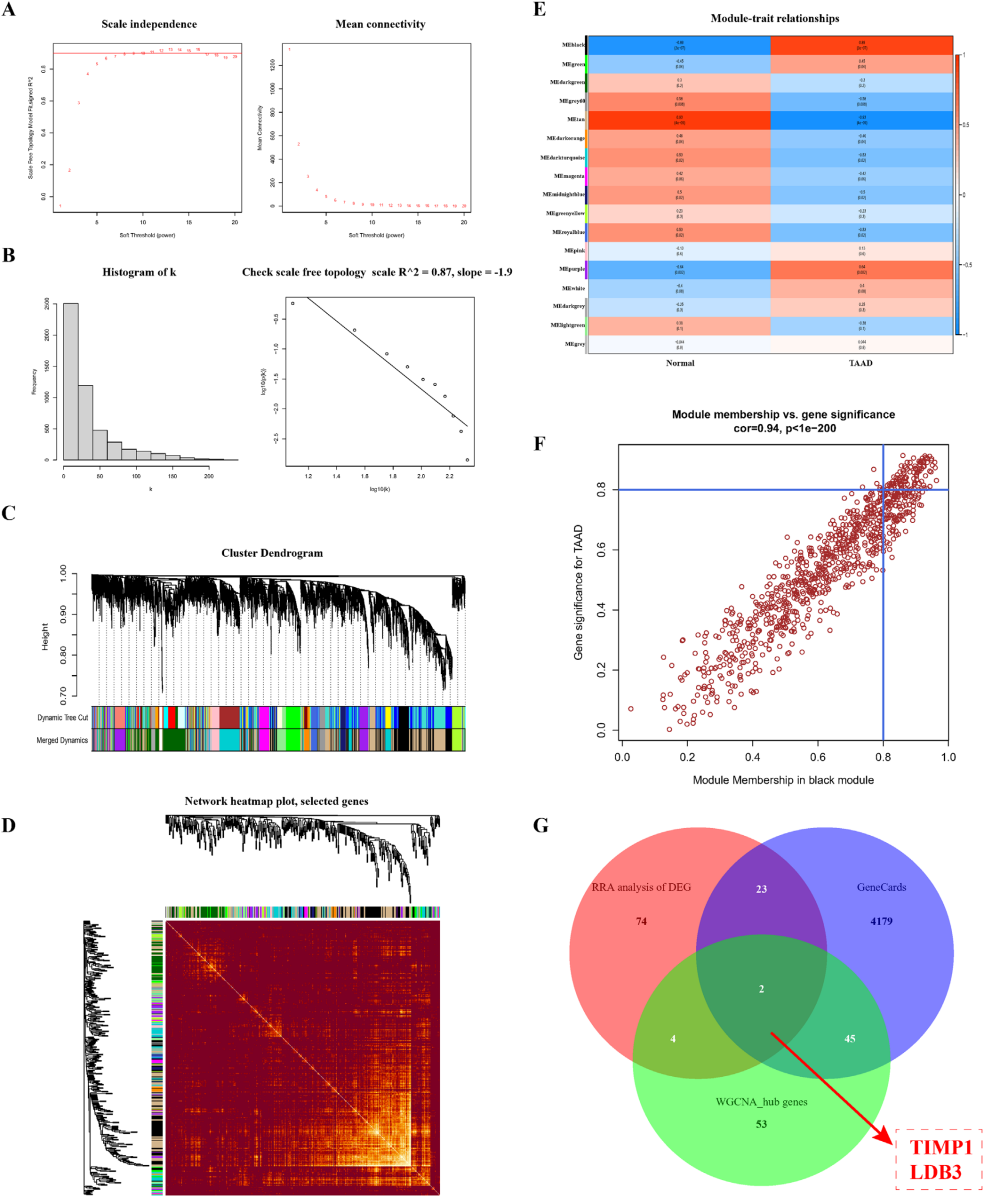
Weighted gene coexpression network analysis of the GSE153434 dataset and Venn diagram to obtain two crucial genes. (A) A soft threshold power value of 8 was used, which is the optimum option. (B) Demonstration of scale‐free network validation with a soft threshold of 8. (C) By clustering genes with strong correlations into the same module, different modules were generated. Different modules are displayed in different colors. (D) Network heatmap showing branching of overall genes associated with modules in a hierarchical clustering dendrogram. (E) Analysis of the correlation between each module and TAAD. (F) The black module was significantly positively correlated with TAAD (correlation coefficient = 0.94, *p* < 0.001). (G) Venn diagram showing the intersection of DEGs obtained after RRA analysis, the WGCNA hub genes, and the TAAD‐related genes in GeneCards, finally yielding TIMP1 and LDB3 as the crucial genes.

WGCNA was conducted on the GSE153434 dataset. The soft threshold power value was set to eight for subsequent analysis (Figure 2A,B). After merging similar modules, dendrograms and heatmaps were used to demonstrate the similarity of the quantified modules according to their correlations (Figure 2C,D). According to the module‐feature association analysis, multiple modules were associated with TAAD; the black module was positively correlated with TAAD and had the strongest correlation (correlation coefficient = 0.94, *p* < 0.001) (Figure 2E). Then, according to gene significance for TAAD > 0.8 and module membership in the black module > 0.8, 104 genes were selected as WGCNA hub genes for subsequent analysis (Figure 2F; File S9). Finally, we intersected the DEGs obtained after RRA analysis, the WGCNA hub genes, and the TAAD‐related genes in GeneCards to obtain two crucial genes, TIMP1 and LDB3 (Figure 2G).

### 3.3 Single‐Cell Sequencing Data Analysis

**FIGURE 3:**
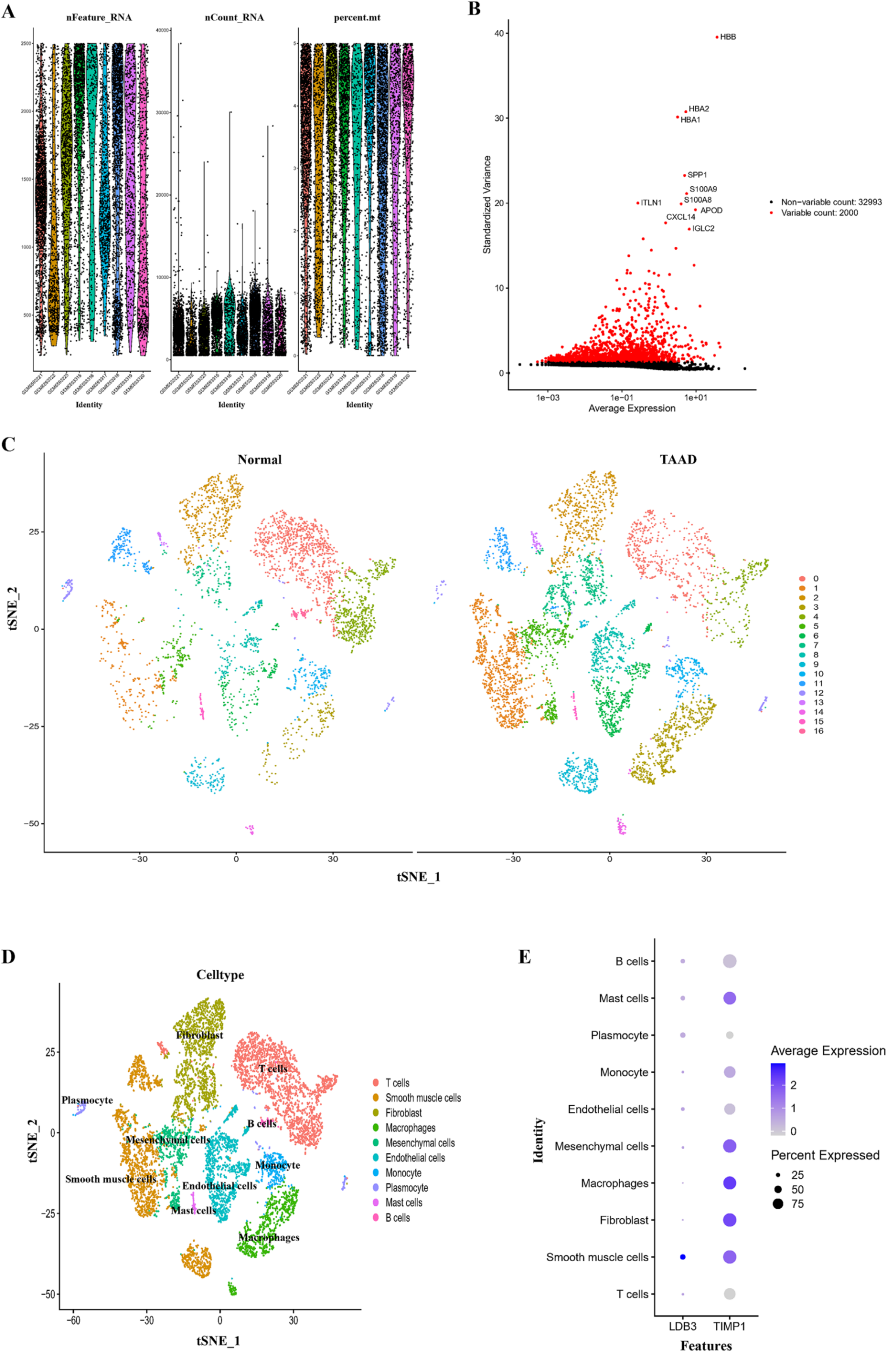
Single‐cell sequencing analysis in the GSE213740 dataset. (A) Cell sample quality was assured by the analysis of three metrics: RNA count, gene count, and mitochondrial gene percentage. (B) The 2000 most highly variable genes are shown in red, with the top 10 highly variable genes marked. (C) Dimensionality reduction and clustering analysis were performed on the cells in the dataset, and the clustering of the TAAD and control groups is displayed in the ‘tSNE’ diagram. (D) Annotation of the clustered cells, including T cells, smooth muscle cells, fibroblasts, macrophages, mesenchymal cells, endothelial cells, monocytes, plasmocytes, giant cells, and B cells. (E) Expression of TIMP1 and LDB3 in cell clusters.

We downloaded the GSE213740 scRNA‐seq dataset from the GEO database to investigate the expression of TIMP1 and LDB3 in different groups of cells in the aortic wall of TAAD patients. To assure the quality of the cell samples used in the study, we first performed quality control on the dataset by removing poor‐quality cells and genes (Figure 3A). The top 2000 genes with high variability were then identified, and the top 10 genes were labeled (Figure 3B). Cells were classified into 17 clusters after the normalization of the dataset and PC analysis (Figure 3C). We then annotated the cells according to the expression of each cluster marker gene (Figure 3D). A detailed list of marker genes is presented in File S10. As shown in Figure 3E, TIMP1 was mainly expressed in macrophages, fibroblasts, mesenchymal cells, and smooth muscle cells, while LDB3 was significantly expressed in smooth muscle cells.

### 3.4 Pseudotemporal Analysis and Functional Enrichment Analysis

**FIGURE 4:**
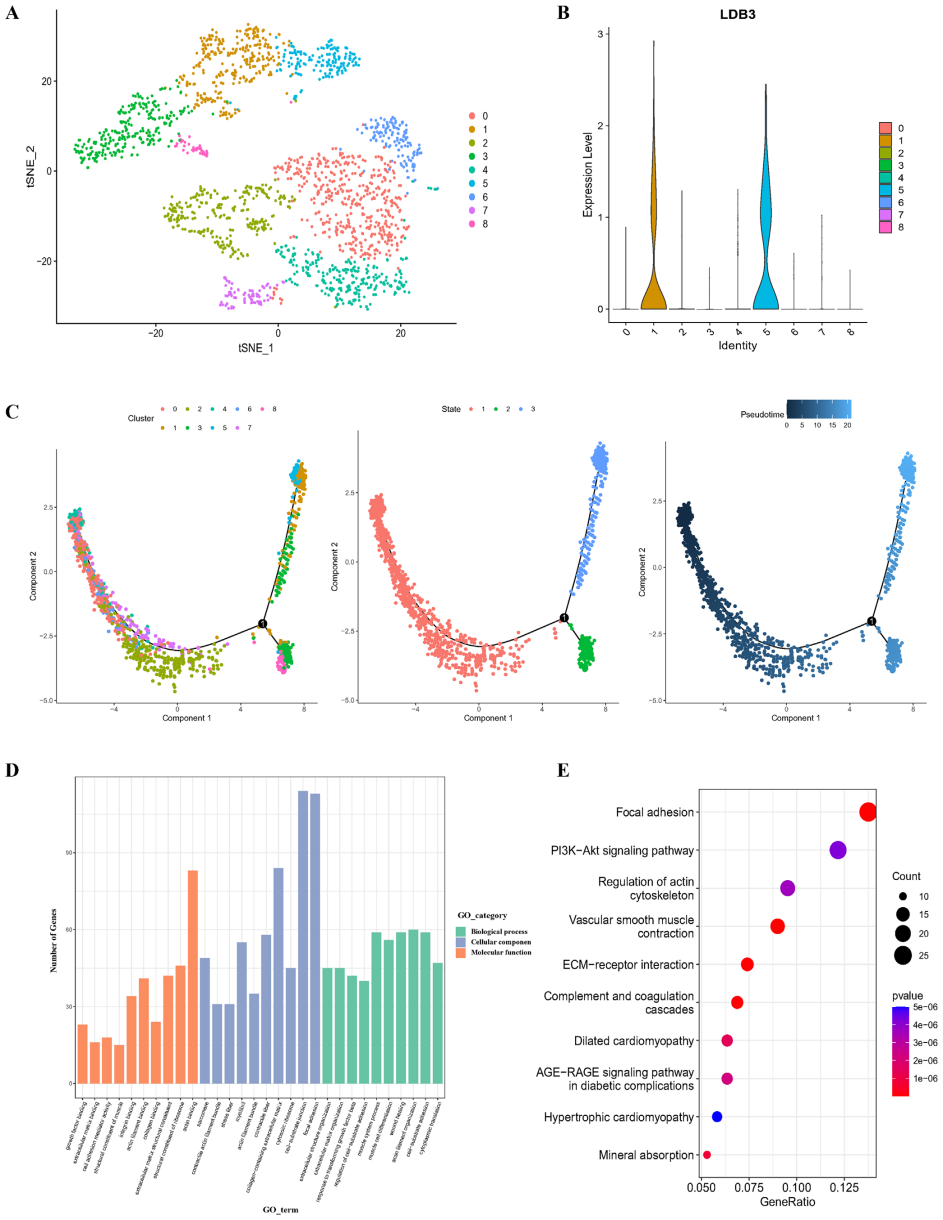
Pseudochronological analysis and pathway enrichment analysis of smooth muscle cells in the GSE213740 dataset. (A) Smooth muscle cells in the dataset were extracted and reclustered into nine clusters. (B) Expression analysis of LDB3 in smooth muscle cells from the GSE213740 dataset. (C) Pseudotime analysis of smooth muscle cells. The three images in the illustration show the three different differentiation states based on the aggregation of nine cell clusters and the differences in the time series of cell differentiation. (D) GO enrichment analysis of significant DEGs between cell clusters 1 and 5 (with considerable LDB3 expression) and the remaining seven cell clusters (with insignificant LDB3 expression). (E) KEGG enrichment analysis based on significantly differentially expressed genes between cell clusters 1 and 5 and the remaining seven cell clusters.

Our scRNA‐seq analysis indicated that TIMP1 was significantly expressed in the aortic wall of TAAD patients, and its role in TAAD was also reported in the study of Corbitt, Vianello and Ishii et al. [17, 18, 19]. However, the mechanism of LDB3 activity in the TAAD aortic wall has yet to be investigated. According to our analysis, LDB3 is mainly expressed in smooth muscle cells, so we extracted the smooth muscle cells in the dataset separately and reclustered them into nine clusters (Figure 4A). As shown in Figure 4B, LDB3 was significantly expressed mainly in clusters 1 and 5. We subsequently performed pseudotime analysis to predict the cell trajectories of smooth muscle cells (Figure 4C). In the pseudotime analysis, one root and two branches were identified, and smooth muscle cells were classified into three distinct differentiation states. Notably, clusters 0, 2, 6 and 7 were mainly in the early stage of differentiation, clusters 8 and 4 were in an intermediate phase, and clusters 1, 5 and 3 were in the end stage. This indicates that the expression of LDB3 may be higher in the late stages of aortic wall formation. This is consistent with previous studies of LDB3 in skeletal and cardiac muscle showing that it is barely detectable during embryogenesis but is strikingly induced postnatally [20]. Finally, we screened the significantly differentially expressed genes of clusters 1 and 5 with substantial LDB3 expression and the other 7 clusters without significant LDB3 expression and performed GO and KEGG enrichment analyses to investigate the mechanism of LDB3 in smooth muscle cells. The screened significantly differentially expressed genes are shown in File S11. Biological processes, cellular components, and molecular functions were among the GO analysis results (Figure 4D). The set of identified genes is enriched mostly in biological processes such as actin binding and actin filament binding. Cellular components included focal adhesion and cell–substrate junctions. The molecular functions mainly included cell–substrate adhesion and actin filament organization. Focal adhesion, phosphatidylinositol 3‐kinase/protein kinase B (PI3K/Akt) signalling pathway, regulation of the actin cytoskeleton, contraction of vascular smooth muscle and extracellular matrix (ECM) receptor interaction were among the enriched pathways in the KEGG pathway enrichment analysis (Figure 4E).

### 3.5 Validation of TIMP1 and LDB3 in GEO Datasets

We next validated the expression of TIMP1 and LDB3 in the following three datasets: GSE190635, GSE52093 and GSE153434. The expression of TIMP1 was significantly upregulated in TAAD samples relative to controls (*p* < 0.05), while LDB3 expression was significantly downregulated (*p* < 0.05) (File S12). Simultaneously, the areas under the curve (AUCs) of TIMP1 and LDB3 in all three datasets were > 0.8, implying that these two genes could be accurate biomarkers for TAAD patients.

### 3.6 LDB3‐Related SNPs Analysis in the UK Biobank

**FIGURE 5:**
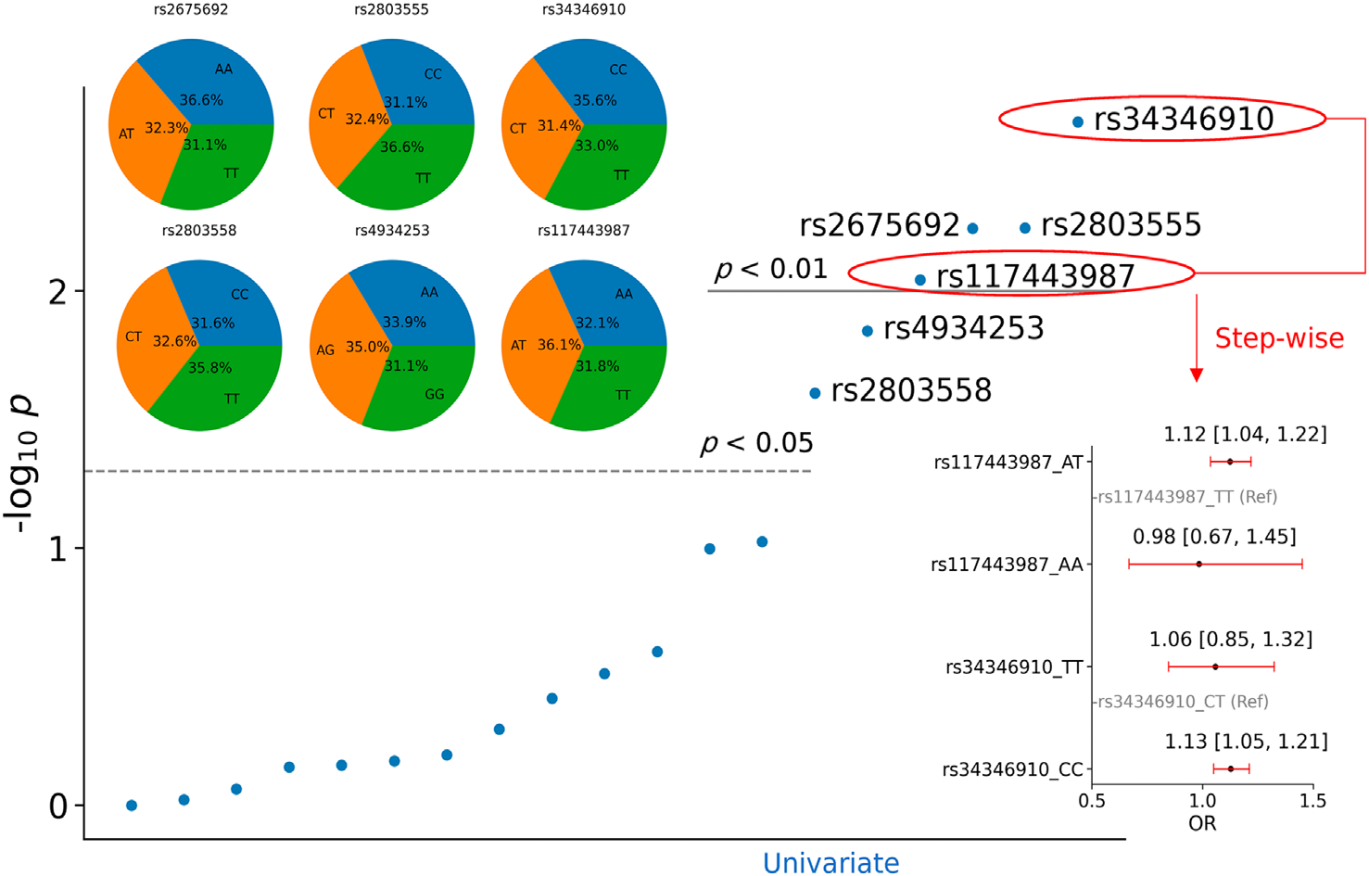
LDB3 SNPs analysis in the UK Biobank. Univariate (19 SNPs) and multivariate (rs34346901 and rs117443987) associations between SNPs and aortic aneurysm and dissection. Six SNPs were significantly related, and the relative ratios of each genotype for aortic aneurysm and dissection are shown in the pie charts.

We found 4577 individuals (9.39%) in the UK Biobank who reported aortic aneurysm and dissection. Figure 5 displays the univariate and multivariate associations between SNPs and aortic aneurysm and dissection. The univariate Chi‐square test (*χ* ^2^) detected two SNPs with *p*‐values < 0.05, namely, rs2803558 (*χ* ^2^ = 7.383, *p* = 0.025) and rs4934253 (*χ* ^2^ = 8.497, *p* = 0.014), and four SNPs with *p*‐values < 0.01, namely, rs117443987 (*χ* ^2^ = 9.415, *p* = 0.009), rs2675692 (*χ* ^2^ = 10.337, *p* = 0.006), rs2803555 (*χ* ^2^ = 10.348, *p* = 0.006) and rs34346910 (*χ* ^2^ = 12.243, *p* = 0.002). The relative ratios of each genotype of aortic aneurysm and dissection are shown in the pie charts. For example, regarding rs34346901, ‘CT’ accounted for 31.4% of aortic aneurysms, normalized by the population ratio of each genotype. Compared to ‘CT’, ‘CC’ increased the likelihood of aortic aneurysm and dissection by 13.41% (95% CI: 5.63%, 21.78%) in rs34346901. Moreover, multiple logistic regression showed that rs34346901 (‘CC’ vs. ‘CT’: OR = 1.13, 95% CI = [1.04, 1.21], *p* = 0.001) and rs117443987 (‘AT’ vs. ‘TT’: OR = 1.12, 95% CI = [1.04, 1.22], *p* = 0.005) significantly contributed to aortic aneurysm and dissection.

### 3.7 Validation of LDB3 in Human Samples

**FIGURE 6:**
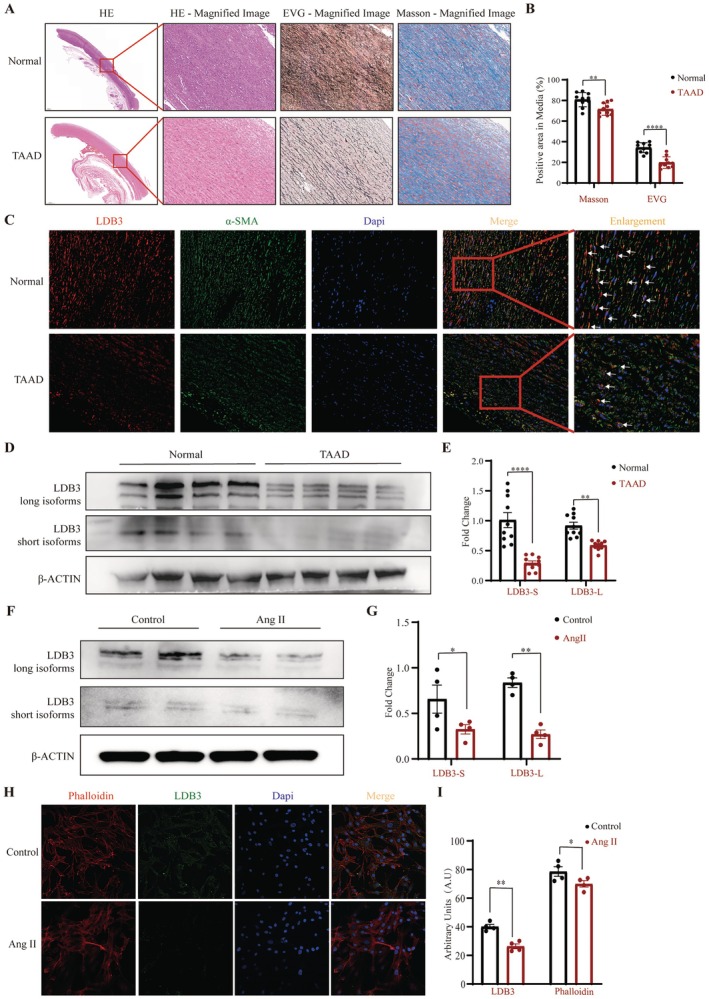
Validation of LDB3 in human samples and upregulation of LDB3 by Ang II stimulation in HA‐VSMC. (A) Histology staining of TAAD and normal tissue. HE, Masson's trichrome staining, and EVG staining were used to reveal the differences in cellularity, collagen fibers, and elastic fibers in ascending aorta sections of TAAD and normal tissues. (B) Tissue collagen and elastin quantification (*n* = 10). (C) Immunofluorescence staining showed that the expression of LDB3 was significantly decreased in TAAD vascular smooth muscle cells. (D, E) Long and short isoforms of LDB3 were expressed in TAAD aortic wall samples, and Western blot verification revealed that both long and short LDB3 expressions were significantly downregulated in TAAD compared to normal samples (*n* = 10). (F, G) Ang II induced HA‐VSMC significant bands of LDB3 and quantification analysis. (H, I) Immunofluorescence staining of LDB3 and phalloidin in HA‐VSMC after Ang II treatment and quantification analysis (*n* = 4). *p < 0.05, **p < 0.01, ****p < 0.0001 in comparisons of two groups as indicated or compared with the corresponding control.

We carried out histological and immunofluorescence labeling on the collected human samples to verify the expression of LDB3 in the TAAD aortic wall tissue. In the HE staining images, smooth muscle cells in TAAD tissue lost their normal regular arrangement and cell morphology, and the intima of the arterial wall was significantly thickened. The collagen and elastic fibers in the TAAD aortic wall tissue were noticeably fragmented, and their arrangement was disorganized, as shown in the Masson and EVG staining images (Figure 6A,B). Immunofluorescence images showed that the expression of LDB3 in the TAAD aortic wall tissue was significantly lower than that of controls. Then, immunofluorescence staining was used to localize the expression of the marker gene α‐SMA in vascular smooth muscle cells. LDB3 was mostly expressed in vascular smooth muscle cells, and compared with the control group, the expression of LDB3 in vascular smooth muscle was significantly decreased in TAAD (Figure 6C). Finally, using western blot analysis, we further investigated the variation of LDB3 expression in human aortic wall tissues. It was evident that the expression of both the long and short isoforms of LDB3 was significantly reduced in TAAD (Figure 6D,E). This is consistent with the findings of our data analysis.

### 3.8 Upregulation of LDB3 by Ang II in HA‐VSMC In Vitro

In order to exclude the reason for smooth muscle cell reduction in human aortic dissection tissue, we further cultured the human smooth muscle cell line HA‐VSMC in vitro. WB and immunofluorescent staining were performed after 100 nM Ang II stimulation for 24 h. We found that Ang II could decrease the expression of both short and long LDB3 isoforms (Figure 6F,G). Also, immunofluorescent staining showed a reduction of LDB3 and F‐actin (Phalloidin), LDB3 was more likely located at intercellular connections (Figure 6H,I).

### 3.9 Validation of LDB3 Expression in Ang II‐Induced Aortic Dissection Aneurysm Models

**FIGURE 7:**
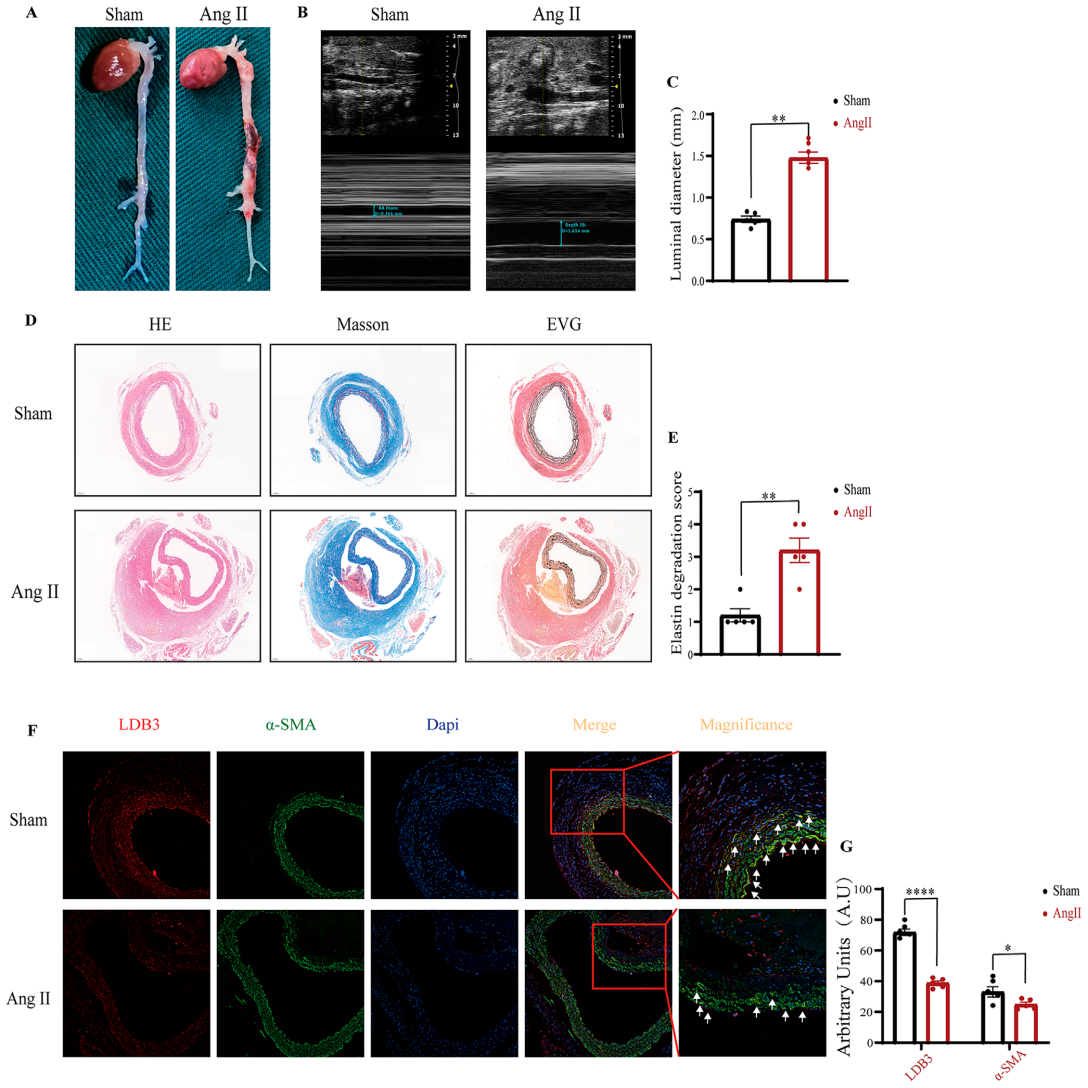
Validation of LDB3 expression in Ang II‐induced aortic dissection aneurysm models. (A) Representative photographs showing macroscopic features of dissection aneurysms induced by Ang II. (B, C) Representative ultrasound images of aortic dimension in two groups and quantification analysis (*n* = 5). (D, E) HE, Masson's trichrome staining, and EVG staining and elastin degradation score analysis (*n* = 5). (F, G) Immunofluorescence staining of LDB3 in Ang II‐induced aortic dissection aneurysm and its quantification analysis (*n* = 5). *p < 0.05, **p < 0.01, ****p < 0.0001 in comparisons of two groups as indicated or compared to the corresponding control.

Ang II induction of aortic dissection and aneurysm is associated with vascular thinning [21]. We, therefore, used an Ang II‐induced mouse model to validate the expression of LDB3. After 4 weeks, mice were found to have significant aneurysm formation (Figure 7A), and vascular Doppler ultrasound imaging showed an increase in aortal diameters (Figure 7B,C). HE and Masson staining significantly showed aortic dissection formation in the Ang II group, and elastin degradation was obvious (Figure 7D,E). Finally, immunofluorescent staining showed that LDB3 was significantly decreased in the Ang II‐induced mouse model and co‐located in SMC as well (Figure 7F,G).

## 4 Discussion

AD is a critical cardiovascular disease characterized by progressive local dilation of the vessel and an ever‐increasing risk of rupture, with high mortality. Media rupture and intima tears occur due to the combined effects of aortic wall stress and abnormalities in the interlayers of the aortic wall [22]. After penetration by blood and the resulting split in the aortic wall, a false lumen emerges during the formation of the mesosphere, separated from the true lumen by a dissection membrane [23]. Asymptomatic ascending aortic thoracic aneurysms are classified as TAAD when they involve the ascending aortic thoracic tract and arch; this is the most severe type [24]. Although many studies investigate the pathogenesis of aortic dissection, little is known about the mechanism that is responsible for its development and progression. In this study, we screened TAAD‐related hub genes by combining DEG screening, WGCNA, and the GeneCards database.

Bioinformatics‐based microarray analysis of gene expression has been widely used to identify disease‐related genes. There is widespread recognition that inflammation of the aortic wall, degradation of the ECM, dysfunction of smooth muscle cells, and remodeling of the arterial wall are the main causes of compromised aortic function. Some emerging studies have shown some underlying pathways involved in hub genes and AD and have mainly focused on the immune response [25, 26]. TIMP1 and LDB3 were ultimately isolated by WGCNA and GeneCards database interaction analyses. Further evaluation of these core genes’ functional and transcriptional characteristics was performed by combining them with single‐cell RNA sequencing.

Endogenous TIMPs regulate matrix metalloproteinase (MMP) expression. MMPs are a family of proteolytic enzymes that degrade the basement membrane and the ECM [27]. To maintain the homeostasis of the vascular wall, MMP and plasminogen activator–plasmin system activity should be balanced with the concentration of TIMPs [28]. In AAA and AD pathologies, abnormal MMP/TIMP ratios can cause excessive ECM breakdown. The multifunctionality of TIMP1 is largely based on its two‐domain structure and comprises the canonical antiproteolytic function mediated via the N‐terminal domain [29]. TIMP1 has been verified to be differentially expressed in aortic tissue and has been identified as a potential plasma biomarker of AD [18]. Since its importance and function are well recognized, we did not perform further validation of TIMP1 in this study.

LDB3 is a highly conserved protein containing a PDZ domain at the NH2 terminus and a LIM domain or domains at the COOH terminus. It is required for the maintenance of the *Z*‐line during muscle contraction and was previously thought to be expressed in striated muscle, mainly skeletal and cardiac muscle [30]. Mutation and aberrant splicing of LDB3 were demonstrated in dilated cardiomyopathy [31] and myotonic dystrophy type 1 [32]. Mutated LDB3 causes cytoskeleton disorganization in cardiomyocytes. We gathered and analyzed all reported SNPs that were pathogenic or associated with LDB3 dysfunction in the UK Biobank. The results yielded 6 SNPs associated with aortic aneurysm and dissection.

There are two long LDB3 isoforms in skeletal and cardiac muscle, each of which has three C‐terminal LIM domains that may function in *Z*‐line structure and in signal transduction. In addition, a short isoform without LIM domains is expressed and mainly localizes to the *Z*‐line [20]. In this study, aortic wall scRNA‐seq analysis and immunofluorescence staining were performed and showed that LDB3 also localized to human aortic smooth muscle cells. To further examine the differential expression of LDB3, western blotting and immunofluorescence fluorescent staining were performed and showed a significant decrease in LDB3 expression in aortic dissection tissue. Meanwhile, the experimental results of the HA‐VSMCs cell line stimulated by Ang II indicate that the downregulation of LDB3 expression is independent of the change in the total number of vascular smooth muscle cells in the diseased tissue. Additionally, the smooth muscle cell *Z*‐line structure was absent from the diseased aortic tunica media layer, as shown in (Figure 6C). We detected both short and long isoforms of LDB3 expression by western blotting, and the expression of all the isoforms was decreased in aortic dissection tissue. The short isoform was only weakly detected in the diseased tissue, which is consistent with the *Z*‐line absence shown by immunofluorescence staining. Interestingly, we found only two long isoforms in the normal aortic wall, as reported in skeletal and cardiac muscle, while there were three bands of LDB3 in the diseased aortic wall (Figure 6D). This result suggested that there may be an LDB3 splicing or isoform that participates in the occurrence of AD. As discovered in dilated cardiomyopathy, LDB3 may participate in *Z*‐line maintenance in the tunica media layer of the aorta, and its alteration or aberrant splicing may play a major role in the pathogenesis of TAAD. Furthermore, whether the mutation of LDB3 is associated with TAAD occurrence also needs to be determined.

## 5 Conclusion

Using bioinformatics approaches, we systematically discovered two important hub genes (TIMP1 and LDB3). In combination with scRNA sequencing, we evaluated TIMP1 and LDB3 expression and localization and identified LDB3‐associated biological processes and pathways. Furthermore, six LDB3‐SNPs were proven to be associated with aortic aneurysm and dissection. LDB3 expression and localization were validated to be downregulated in human aortic wall tissues and the Ang II‐induced mouse model. LDB3 was also reduced by Ang II stimulation in HA‐VSMC in vitro. In summary, LDB3 was newly identified and validated in our study to have an important role in TAAD pathogenesis.

## Author Contributions

**Xinyi Liu:** resources (equal), software (equal), visualization (equal), writing – original draft (equal). **Xing Liu:** validation (equal), writing – original draft (equal). **Bin Wan:** investigation (equal), methodology (equal). **Yipeng Ge:** conceptualization (equal), funding acquisition (equal). **Haiou Hu:** data curation (equal), formal analysis (equal). **Hong Yu:** project administration (equal). **Meng Zhao:** software (equal), supervision (equal). **Huadong Li:** conceptualization (equal), project administration (equal). **Junming Zhu:** conceptualization (equal), formal analysis (equal), funding acquisition (equal), methodology (equal).

## Ethics Statement

This study was approved by the Ethics Committee of The Union Hospital of Hubei Province (0497).

## Consent

All participants in this study provided informed consent.

## Conflicts of Interest

The authors declare no conflicts of interest.

## Supporting information


Data S1

## Data Availability

The datasets presented in this study are available in online repositories. The repository names and accession numbers can be found in the article/Data S1.
