# Supplementary material for: Identification of the Hub Gene LDB3 in Stanford Type A Aortic Dissection Based on Comprehensive Bioinformatics Analysis
Source: J Cell Mol Med. 2025 Mar 18;29(6):e70471. doi: 10.1111/jcmm.70471 (PMC11916769; doi:10.1111/jcmm.70471)
Supplement: Supplementary file 1 — Data S1 [file JCMM-29-e70471-s001.zip › Supplementary File 1, 3, 4, 7, and 12.docx]

**Supplementary File 1. TAAD datasets from several GEO datasets summarized.**

| **Series** | **Platform** | **GeneChip** | **Normal** | **TAAD** | **Year** |
| --- | --- | --- | --- | --- | --- |
| GSE52093 | GPL10558 | Illumina HumanHT-12 V4.0 expression beadchip | 5 | 7 | 2015 |
| GSE190635 | GPL570 | [HG-U133_Plus_2] Affymetrix Human Genome U133 Plus 2.0 Array | 4 | 4 | 2021 |
| GSE153434 | GPL20795 | HiSeq X Ten (Homo sapiens) | 10 | 10 | 2020 |
| GSE213740 | GPL18573 | Illumina NextSeq 500 (Homo sapiens) | 3 | 6 | 2022 |

**Supplementary File 3. Preoperative characteristics of the study population**

| **Variable** | | **Normal** | **TAAD** |
| --- | --- | --- | --- |
| N  **Patient characteristics**  Age (years)  Male  Height (cm)  Weight (Kg)  **Medical history**  Hypertension  Diabetes  **Laboratory parameters**  Haemoglobin (g/L)  Creatinine (μmol/L)  Admission glucose (mmol/L)  APTT (s)  CK-MB (ng/ml)  **Imaging parameters**  EF (%) | | 10  63(55-67)  4(40.0%)  167.3 ± 4.5  70.1 ± 10.4  3(30.0%)  2(20.0%)  121.7 ± 16.1  89.5 ± 42.3  5.1 ± 3.1  33.1(27.6-35.8)  2.1 ± 2.8  32.6 ± 5.1 | 10  56(51-59)  5(50.0%)  168.5 ± 5.5  72.1 ± 11.9  7(70.0%)  1(10.0%)  115.6 ± 17.3  91.3 ± 40.5  5.7 ± 2.8  36.1(34.6-39.3)  2.4 ± 2.1  60.8 ± 4.3 |
|  |  | |  |

**Supplementary File 4. Volcano plots of DEGs and heatmaps for the GSE52093 and GSE190635 datasets**

**
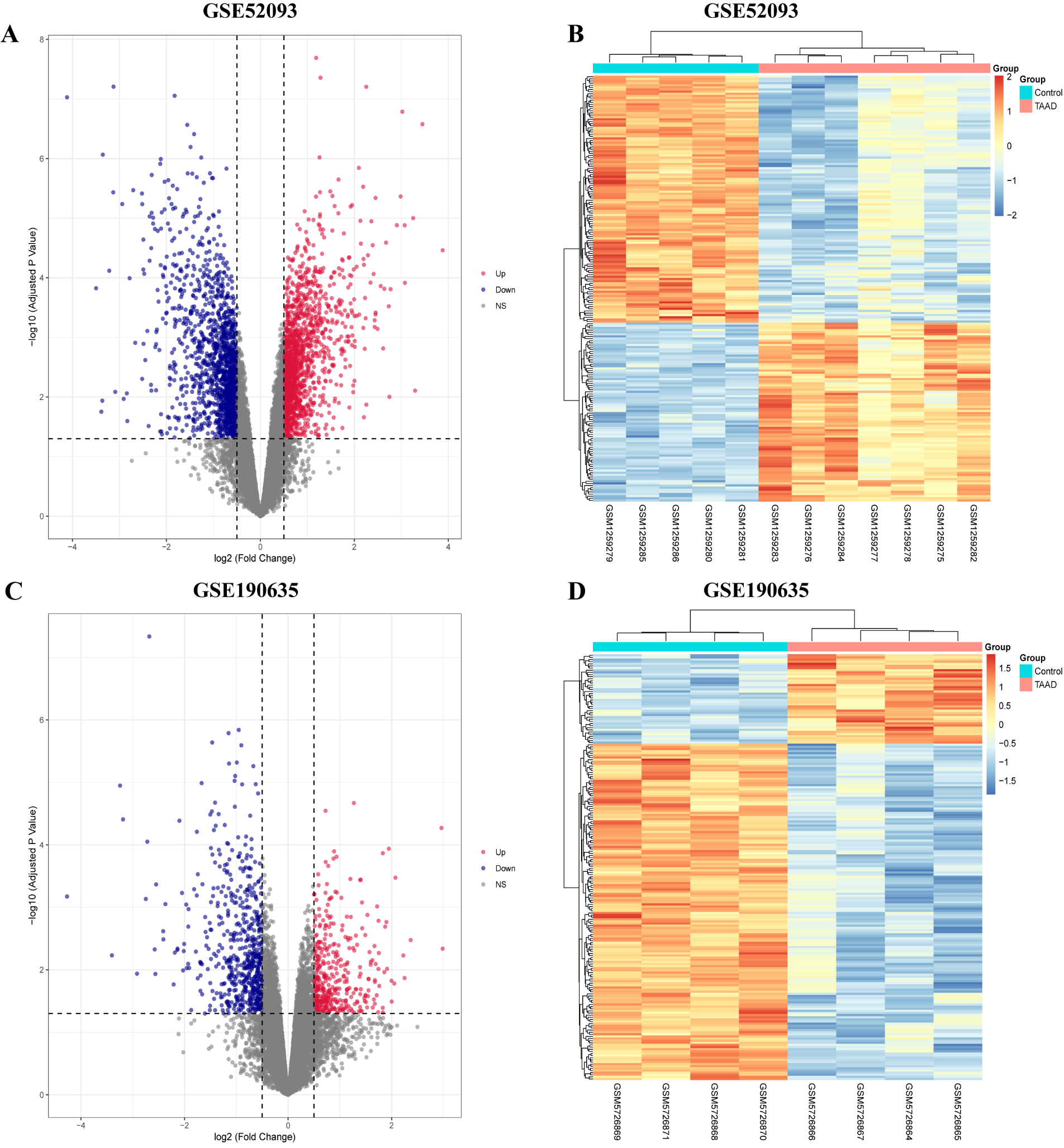
**

**(A and C)** Volcano plots of DEGs from GSE52093 and GSE190635. Red dots denote upregulated genes, blue dots denote downregulated genes, and gray dots denote genes with no significant difference in expression. **(B and D)** Heatmaps of DEGs from GSE52093 and GSE190635. Red represents upregulated DEGs, blue represents downregulated DEGs.

**Supplementary File 7. RRA anylasis Rcode**

library(RobustRankAggreg)
library(tidyverse)

##Screening for up- and down-regulated genes in each dataset, DEG indicates significantly differentially expressed genes.

glist_up <- list()

glist_down <- list()

for(i in 1:2){

glist_up[[i]] <- get(paste0(GSEs[i],"_DEG")) %>% filter(logFC > 0) %>% .[[1]]

glist_down[[i]] <- get(paste0(GSEs[i],"_DEG")) %>% filter(logFC < 0) %>% .[[1]]

}

## Perform RRA analysis

res_RRA_up <- aggregateRanks(glist_up)

res_RRA_down <- aggregateRanks(glist_down)

## Combine the results of upregulation and downregulation

all_signi_result <- bind_rows(res_RRA_up %>% mutate(direction = "up"),

res_RRA_down %>% mutate(direction = "down")) %>%

filter(Score < 0.05) %>%

rownames_to_column(var = "Gene Symbol") %>%

write_tsv("RRA analysis of differentially expressed genes.tsv")

**Supplementary File 12. TIMP1 and LDB3 validation in datasets from the GEO database.**

**
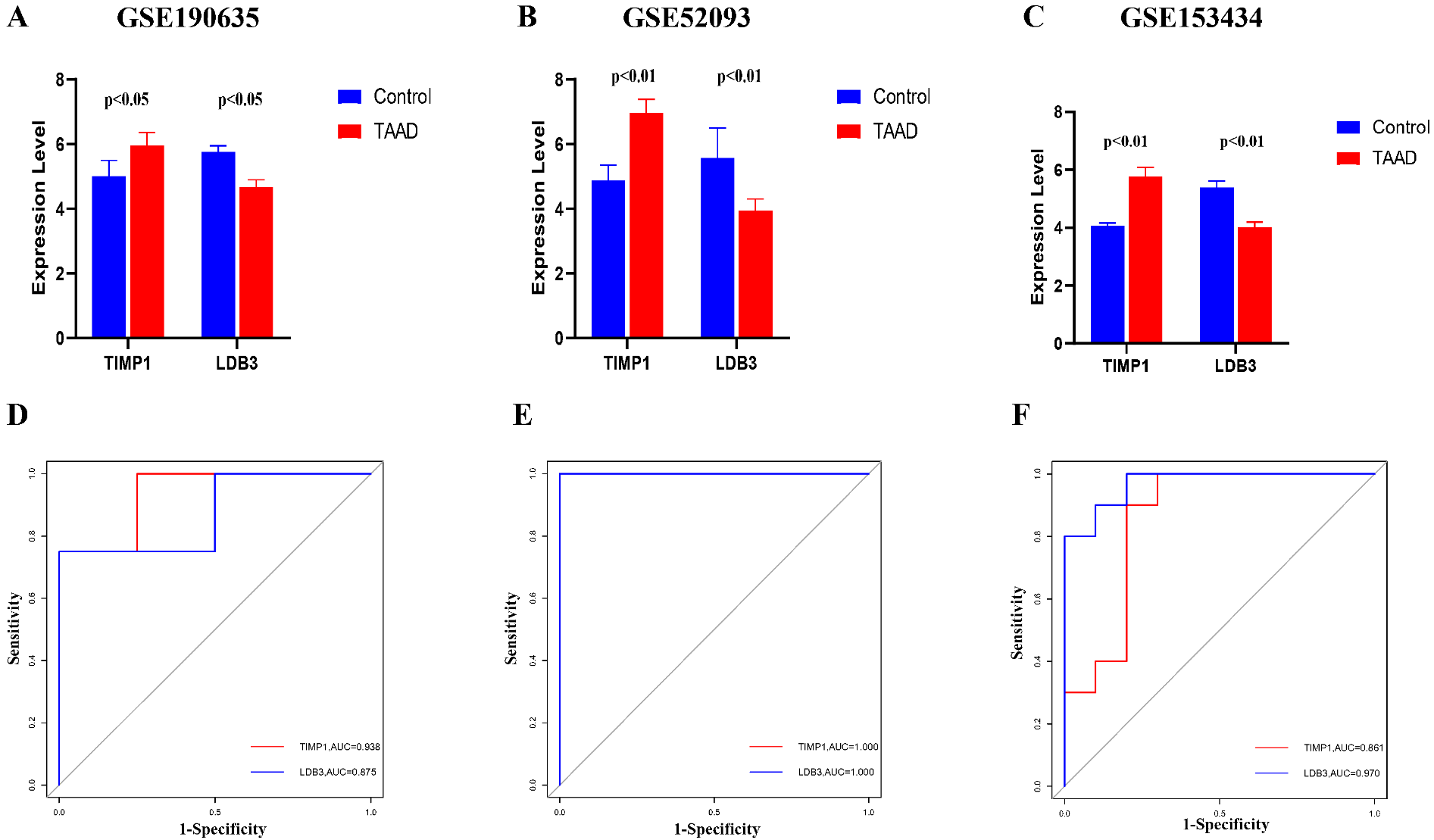
**

**(A-C)** TIMP1 and LDB3 expression levels in the three datasets GSE190635, GSE52093, and GSE153434. (D-F) TIMP1 and LDB3 receiver operating characteristic (ROC) curves in the three datasets GSE190635, GSE52093, and GSE153434 demonstrate their diagnostic value.
